# Physical and Biochemical Determinants of Endocrine Fate in 3D Suspension Culture Based Stem Cell-Derived β-Cell Differentiation

**DOI:** 10.3390/cells15171600

**Published:** 2026-09-02

**Authors:** Roberto Castro-Gutierrez, Ali H. Shilleh, Jessie M. Barra, Shane P. M. Williams, Balachandar Nedumaran, Taylor M. Triolo, Matthias Hebrok, Holger A. Russ

**Affiliations:** 1 Diabetes Institute, University of Florida, Gainesville, FL 32610, USA; 2 Department of Pharmacology and Therapeutics, University of Florida, Gainesville, FL 32610, USA; 3 UCSF Diabetes Center, University of California San Francisco, San Francisco, CA 94134, USA; 4 Barbara Davis Center for Diabetes, Department of Pediatrics, University of Colorado Anschutz Medical Campus, Aurora, CO 80045, USA; 5 Oxford Centre for Diabetes, Endocrinology and Metabolism, Radcliffe Department of Medicine, University of Oxford, Churchill Hospital, Oxford OX3 7LE, UK; 6 Center for Organoid Systems, University Hospital rechts der Isar (MRI), Technical University Munich, 85748 Garching, Germany; 7 Institute for Diabetes Organoid Technology, Helmholtz Munich, Helmholtz Diabetes Center, Ingolstädter Landstraße 1, 85764 Neuherberg, Germany; 8 Munich Institute of Biomedical Engineering (MIBE), Technical University of Munich, 85748 Garching, Germany; 9 German Center for Diabetes Research (DZD), 85764 Neuherberg, Germany

**Keywords:** human pluripotent stem cells, insulin-producing, stem cell-derived beta cells, direct differentiation, diabetes

## Abstract

Efficient and reproducible generation of functional stem cell-derived β cells (sBC) remains a major challenge for basic research and cell replacement therapy, partly due to incomplete understanding of endocrine induction during differentiation and challenges with clinical scale-up. Here, we dissect the individual contributions of commonly used endocrine differentiation factors on pancreatic progenitor maintenance, endocrine commitment, and hormone subset generation in a scalable 3D differentiation system. We demonstrated that starting pluripotent stem cell cluster size is a critical determinant for downstream sBC generation. We also verify that a commonly employed combination of endocrine induction molecules efficiently drives endocrine lineage commitment but yields limited β-cell generation. Detailed analysis of the effects of individual endocrine induction molecules revealed distinct effects: EGF or KGF preserved NKX6.1^+^ progenitors without induction of endocrine differentiation; Notch or BMP inhibition robustly induced endocrine marker expression but concurrently reduced NKX6.1 expression, resulting in predominant generation of glucagon-expressing cells; retinoic acid, thyroid hormone (T3), or TGFβ inhibition maintained high NKX6.1 levels while also promoting efficient insulin^+^ endocrine differentiation. These findings indicate NKX6.1 protein maintenance as a key determinant of human β-cell generation and show that endocrine differentiation factors exert divergent effects on lineage progression.

## 1. Introduction

Replacement of insulin-producing pancreatic β cells holds great promise as a curative therapy for diabetes. Over the past decade, advances in human pluripotent stem cell (hPSC) differentiation have enabled the scalable generation of pancreatic progenitors and stem cell-derived β cells (sBC) that recapitulate key molecular, ultrastructural, and functional features of primary human β cells, including glucose-responsive insulin secretion in vitro and in vivo [1,2,3,4,5,6,7,8,9,10,11,12]. Despite these successes, current differentiation protocols remain limited by incomplete β-cell generation and the frequent emergence of off-target cell types [7,10,11,12]. These limitations highlight critical gaps in our understanding of the developmental signals governing endocrine induction and fate decisions in human systems.

Most contemporary sBC differentiation strategies accurately recapitulate early stages required for pancreatic development, resulting in efficient generation of PDX1^+^ pancreatic foregut endoderm and subsequent bipotent pancreatic progenitors marked by co-expression of PDX1 and NKX6.1. However, the subsequent endocrine induction stage represents a persistent bottleneck for β-cell generation, resulting in only partial generation of a desired insulin-producing cell phenotype [1,2,3,4,5,6,7,8,9,10,11,12].Endocrine differentiation is commonly triggered using combinations of signaling pathway modulators including inhibition of Notch (γ-secretase), TGFβ, and BMP signaling, together with thyroid hormone or retinoic acid activation [7,10,11,12]. This molecule cocktail is derived from the established literature defining key pathways from studying pancreas organogenesis in vivo using mice [13,14,15,16]. However, as these factors are typically applied simultaneously, we lack detailed knowledge on their individual effects on progenitor maintenance, endocrine commitment, and hormone specification in the human context. Beyond lineage generation, clinical translation of sBC imposes additional constraints related to scalability, reproducibility, and manufacturing robustness. Differentiation protocols must be compatible with suspension cultures amendable to up-scaling, yield consistent cellular compositions across multiple batches, and minimize dependence on complex or poorly defined factor combinations [17,18]. A mechanistic understanding of how individual signaling pathways influence fate decisions in a human model during endocrine induction is therefore essential not only for improving differentiation efficiency but also for enabling more robust, scalable manufacturing of sBC products.

Here, utilizing a suspension-based 3D differentiation system [9], we sought to systematically dissect the effects of both cluster size and individual signaling molecules commonly employed during endocrine induction on pancreatic progenitor maintenance, endocrine commitment (NEUROG3 expression), and hormone expression. Using temporal marker analysis, single-factor perturbations, and an insulin reporter hPSC line, we directly compare the capacity of different signals to drive endocrine differentiation while preserving key progenitor features associated with β-cell fate. Our results reveal that endocrine induction and β-cell generation are separable processes and identify maintenance of NKX6.1 protein expression as a critical determinant associated with insulin-producing β-cell generation. Together, these findings provide mechanistic insight into the limitations of current differentiation strategies and establish a framework for rational, scalable optimization of endocrine induction to enhance sBC yield and identity.

## 2. Materials and Methods

### 2.1. Human Stem Cell Culture and sBC Differentiation

Human pluripotent stem (hPSC) Mel1^INS-GFP^ cells (National Institutes of Health (NIH) registry, Bethesda, MD, USA, #0139) [19] or induced pluripotent stem cells [9] were maintained Cultrex (Biotechne, Minneapolis, MN, USA #3434-005-002) in mTeSR+ media (STEMCELL Technologies, Vancouver, Canada #05826). For generation of clusters using microwells, single-cell suspensions were generated by incubating hPSC for 6–8 min in TryPLE (Gibco, Thermo Fisher Scientific, Waltham, MA, USA), followed by quenching with media. Live cells were filtered through a 40 μm mesh to remove potentially confounding cell clusters; single live cells were counted using trypan blue to exclude dead cells and appropriate cell numbers were seeded into AggreWells plates 400 (for 1K clusters) or 800 (all other sizes) (Stem Cell Technology, Vancouver, Canada) in mTESR+ media containing 10 μM ROCK inhibitor Y-27632 (Tocris, Bristol, UK). After 24 hours, formed clusters were transferred to suspension culture plates. Differentiation into stem cell-derived β-like cells was carried out as described (Reprocell #ABBWVS03A-6, #ABBWVDW-1013, and #ABBWBP03N0S-6) [9]. sBC differentiation was based on our published protocol with modifications as outlined below with key experiments using select additions or subtractions from medias as described in the results and figures. Altered differentiation medias are as follows. d9-15: DMEM containing 1:50 N-21 MAX, 1:100 NEAA, 1 mM Sodium Pyruvate, 1:100 GlutaMAX, 10 μg/ml Heparin (Sigma, St. Louis, MO, USA, #H3149-250KU), 2 mM N-Acetyl-L-cysteine (Cysteine) (Sigma, St. Louis, MO, USA, #A9165-25G), 10 μM Zinc sulfate heptahydrate (Zinc) (Sigma, St. Louis, MO, USA, #Z0251-100g), 1× BME, 10 μM ALK5i II RepSox (R&D Systems, Minneapolis, MN, USA, #3742/50), 1 μM 3,3’,5-Triiodo-L-thyronine sodium salt (T3) (Sigma #T6397), 0.5 μM LDN, 1uM Gamma Secretase Inhibitor XX (XXi) (AsisChem, Tianjin, China, #ASIS-0149) and 1:250 1 M NaOH to adjust the pH to ~7.4. d16-30: CMRL (Gibco, Thermo Fisher Scientific, Waltham, MA, USA, #11530-037) containing 1:50 N-21 MAX, 1:100 NEAA, 1:100 GlutaMAX, 10μg/ml Heparin, 2mM Cysteine, 10 μM Zinc, 1× BME, 1 μM T3, 50ug/ml VitC, 1:1000 Trace Elements A (Corning, NY, USA, # 25-021-CI), 1:1000 Trace Elements B (Corning, NY, USA, # 25-022-CI) with 10 μM ALK5i II RepSox and 1:250 NaOH to adjust the pH to ~7.4. All medias with the exception of mTeSR+ also contained 1× PenStrep. 

### 2.2. Flow Cytometry

For sBCs, single-cell suspensions were made by washing clusters with PBS and incubating with 0.05% Trypsin with EDTA (Gibco; Thermo Fisher Scientific, Waltham, MA, USA) at 37 °C for 12–15 min. Single-cell suspensions were quenched with 2% FBS (Corning, Corning, NY, USA) in PBS (Gibco; Thermo Fisher Scientific, Waltham, MA, USA), filtered through a 40 μm cell strainer (Corning, Corning, NY, USA) into FACS 5 ml tubes (Corning, Corning, NY, USA) and in some experiments incubated for 30 min on ice for surface markers/live–dead staining then fixed with 4% paraformaldehyde (Thermo Fisher Scientific, Waltham, MA, USA) for 5–10 min at room temperature and stained in CAS buffer (BioLegend, San Diego, CA, USA) with 0.4% Triton X (Invitrogen, Thermo Fisher Scientific, Waltham, MA, USA, CAS-T) overnight at 4 °C for intracellular markers. After incubation, the cells were washed and resuspended in FACS buffer for analyses on a 5-laser Cytek Aurora. Analysis and graphs were made using FloJo software v10.9 (BD Life Sciences). An antibody list can be found in Supplemental Table S1.

### 2.3. Cluster Size Determination

The image analysis was done using ImageJ (version 1.54s), and we followed the protocol detailed here (https://imagej.net/ij/docs/menus/analyze.html, accessed on 13 July 2026). Outlines of individual clusters were created through the outline function and fill holes function in ImageJ. Cluster sizes were analyzed using the analyze particle tool with circularity from 0.7–1.0 and excluding edges. All clusters found in a frame (10–130) from biological replicates N = 5 were used to determine the average cluster diameter and area per condition.

### 2.4. Immunofluorescence Imaging

sBC clusters were collected in 1.5 mL Eppendorf tubes (Eppendorf SE, Hamburg, Germany) and allowed to gravity-settle for 5 minutes. Clusters were washed with 1× PBS and fixed for 10 minutes in 4% paraformaldehyde. Clusters were placed in 30% sucrose (Sigma-Aldrich, St. Louis, MO, USA) overnight followed by embedding in OCT (Tissue-Tek®; Sakura Finetek USA, Torrance, CA, USA) blocks and frozen in dry ice/ethanol bath before placement at −80 °C for storage. Clusters were sectioned at 6 μm and placed on slides for staining. Sections were blocked/permeabilized with CAS buffer (BioLegend, San Diego, CA, USA) with 0.4% Triton X (CAS-T, Thermo Fisher Scientific, Waltham, MA, USA) for 10 minutes and then stained with primary antibodies overnight at 4 °C in CAS-T buffer. After incubation, the slides were washed and stained with fluorochrome-conjugated secondary antibodies for 2 hours at room temperature in CAS-T buffer. Slides were then mounted with ProLong Gold antifade reagent with DAPI (Invitrogen, Carlsbad, CA, USA) and sealed with cover slips before imaging and analysis on the confocal microscope (Carl Zeiss LSM 800, Jena, Germany) (20× objective). A list of antibodies and dyes used can be found in Supplemental Table S2.

### 2.5. Gene Expression Analysis

Total RNA was isolated with the RNeasy Micro kit (74104; QIAGEN, Hilden, Germany) and reverse-transcribed with the iScript cDNA kit (1708891; Bio-Rad Laboratories, Hercules, CA, USA) as per the manufacturer’s instructions. Quantitative PCR analysis was performed on the Bio-Rad CFX96 Real Time System (Hercules, CA, USA) using TaqMan probes (Supplemental Table S3). CT values were normalized to *GAPDH,* and a one-way ANOVA with multiple comparisons was performed (using the base condition as comparison for Supplemental Figure S1).

### 2.6. Statistical Analysis

Statistical analyses were performed using GraphPad Prism v10.1.0 (GraphPad Software, La Jolla, CA, USA).

## 3. Results

### 3.1. Differentiation Efficiency in Suspension Culture Is Dependent on Initial hPSC Cluster Size

**Figure 1:**
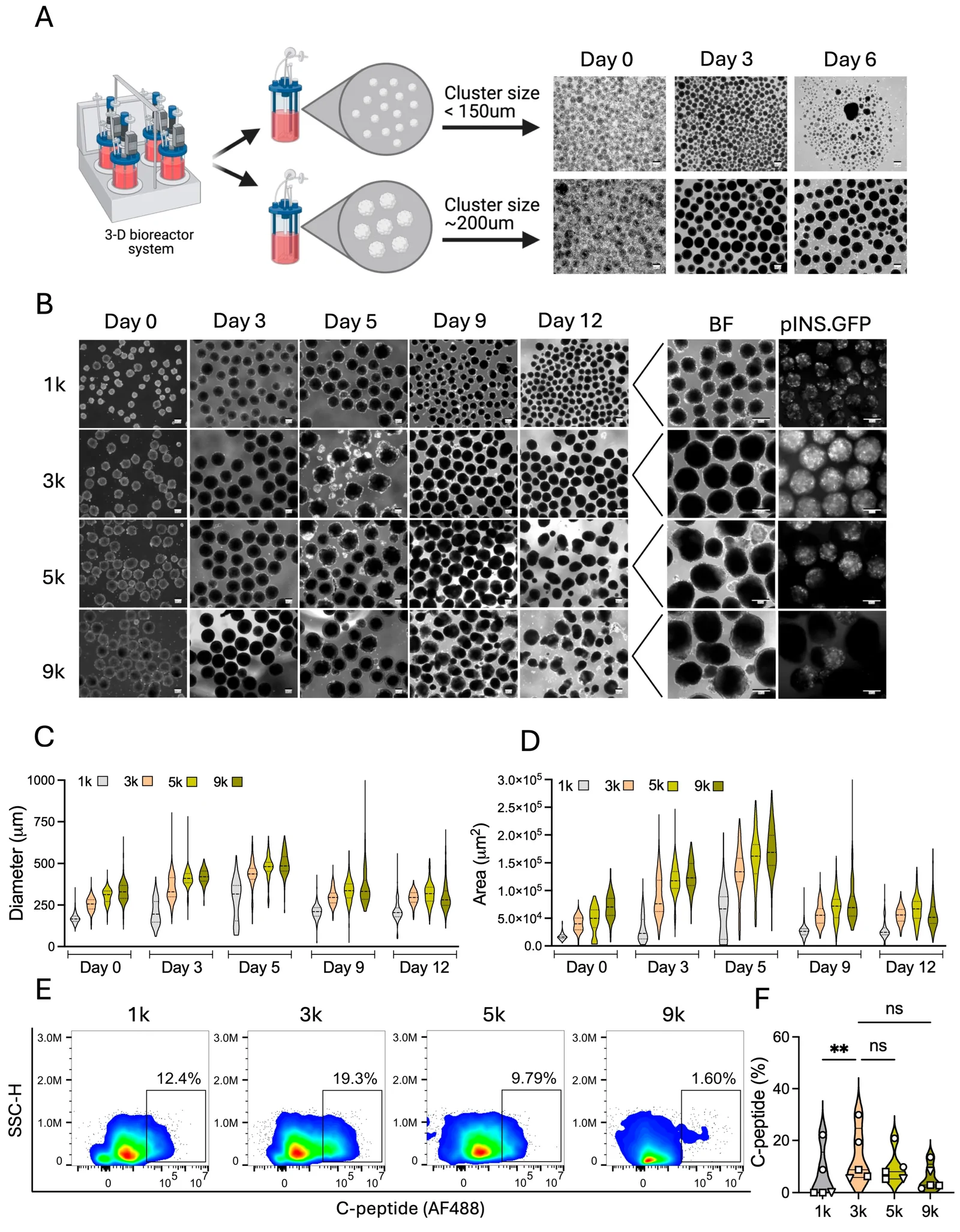
Initial cluster size determines sBC differentiation outcome. (**A**) Schematic of 3D stirrer system sBC differentiation started with different cluster sizes. Representative bright field images on days 0, 3, and 6. Scale bar: 200um. (**B**) Bright field (BF) and pINS.GFP images at subsequent days of sBC differentiation of clusters generated with increasing numbers of cells. Scale bar: 200 μm. Images labeled day 0-day 12 are 4× and images labeled BF or pINS.GFP are 10× (**C**,**D**) Violin plots measuring diameter (**C**) and area (**D**) of clusters generated with different numbers of cells (1K silver, 3K peach, 5K chartreuse, 10K olive) during subsequent stages of sBC differentiation. N= (10–130) clusters per day from N= 5 independent differentiation experiments (IDEs) using both embryonic stem (ES) and induced pluripotent stem cell (iPSC) lines. **(E)** Representative flow cytometry analysis of clusters generated with different numbers of cells for the sBC marker C-peptide on day 12 of differentiation. (**F**) Quantification of the percentage of C-peptide^+^ cells present in clusters generated with different numbers of cells after 12 days of sBC differentiation. Data represents 5 independent direct differentiation experiment, 2 replicates with ESC (white circles), 2 replicates with 1 iPSC (white squares) and 1 replicate with a second iPSC line (white triangles). One-way ANOVA with a multiple comparison test was used to determine statistical significance with ** *p* < 0.01.

To assess how three-dimensional (3D) culture parameters influence differentiation efficiency, we examined the impact of initial hPSC cluster size on downstream pancreatic differentiation outcomes using a suspension culture-based system. We generated hPSC aggregates [9] with diameters smaller than 150 μm or approximately 200 μm by varying seeding density at cluster induction (Figure 1A). Direct differentiation of the cultures resulted in the collapse of smaller clusters by day 6 of differentiation while larger clusters survived readily (Figure 1A). To further understand this size determinant, we utilized microwell plates to generate clusters comprising increasing numbers of cells per cluster and tracked their gross morphology through the early stages of pancreatic direct differentiation (Figure 1B). We demonstrated that differentiation efficiency into insulin-expressing cells was highly sensitive to initial cluster size. Aggregates with 3000 cells (3K) per cluster at induction consistently exhibited improved gross morphology throughout differentiation (Figure 1B). The 3K clusters maintained smooth borders, spherical architecture, and increased on target cell generation as observed by a GFP reporter driven under the endogenous insulin promoter (pINS.GFP) [19] compared to both smaller (1K) and larger (5K or 9K) aggregates, which displayed irregular shapes, increased debris, or evidence of off-target, cystic structures (Figure 1B). Quantitative assessment of cluster diameter and size at key stages of differentiation through early endocrine commitment (day 12) confirmed more consistent cluster growth dynamics and reduced cluster collapse from 3K clusters (Figure 1C,D, Supplementary Tables S4 and S5). Flow cytometry (FC) analysis revealed increased expression of the β-cell marker, C-peptide on day 12 by 3K clusters compared to smaller and larger clusters (Figure 1E,F). These findings suggest that optimal aggregate size is a prerequisite for effective lineage progression in 3D differentiation systems.

### 3.2. Standard Endocrine Induction Promotes Endocrine Differentiation Without Robust β-Cell Fate Commitment

**Figure 2:**
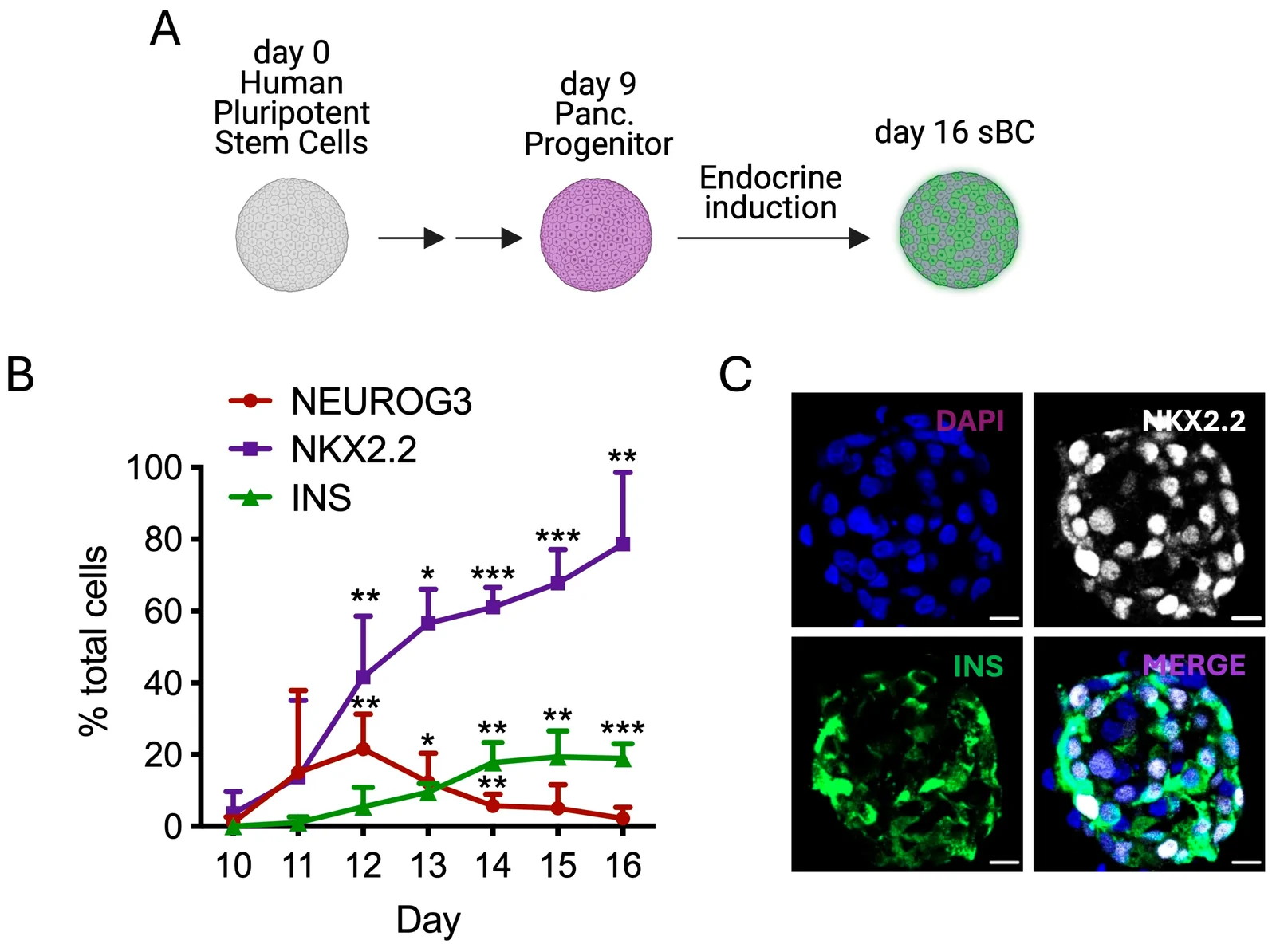
Time course analysis of endocrine cell differentiation during sBC differentiation using a published protocol. (**A**) Schematic of experimental approach to determine expression changes in key markers during standard endocrine cell differentiation. (**B**) Quantification of the percentage of cells expressing the endocrine differentiation proteins NEUROG3 and NKX2.2 and the hormone insulin (INS) from day 10 to day 16 during endocrine commitment measured by immunofluorescence. Data presented as mean +/− standard deviation. Two-way ANOVA with a multiple comparison test with Geisser–Greenhouse correction compared to day 10 data point for each differentiation was used to determine statistical significance * *p* < 0.05; ** *p* < 0.01; *** *p* < 0.001. Data represents 3 independent direct differentiation experiments. (**C**) Representative stain of day 16 sBCs for DAPI (blue), NKX2.2 (white), and insulin (green). Scale bar: 50μm.

To develop a better understanding of the endocrine induction stage during direct differentiation, we assessed protein marker expression associated with early endocrine differentiation induction (NEUROG3), endocrine lineage commitment (NKX2.2) and a β-cell phenotype (insulin) from day 9 to day 16 (Figure 2A) employing an endocrine media containing inhibitors for TGFβ, BMP and gamma secretase (XXi) signaling, as well as activating molecule T3. This molecule cocktail is a well-established combination for the differentiation of endocrine cell types from pancreatic progenitors [1,2,3,4,5,6,7,8,9,10,11,12,20,21,22,23]. Short-lived NEUROG3 expression is robustly detected on day 11–12, while the endocrine lineage marker NKX2.2, which is downstream of NEUROG3, continuously increases, reaching ~80% of all cells on day 16, indicating that most pancreatic progenitors differentiated efficiently towards an endocrine phenotype using this molecule cocktail (Figure 2B,C). However, only a small fraction of approximately 20% of all cells on day 16 expressed insulin, indicating limited on-target commitment to the desired β-cell fate (Figure 2B,C). Next, we asked if endocrine-committed cells that lack insulin expression could be induced to activate an sBC phenotype by re-exposure to the endocrine molecule cocktail. We took advantage of the pINS.GFP reporter to sort pINS.GFP negative and positive cells on day 23 and reaggregated them at 3,000 cells/cluster followed by incubation in the endocrine molecule cocktail for an additional 6 days (Supplemental Figure S2A). While GFP^+^ clusters largely maintained insulin reporter expression, no significant GFP expression was detected in any of the sorted GFP^-^ clusters (Supplemental Figure S2B), demonstrating that re-exposure to endocrine induction media does not result in appreciable generation of sBCs from the endocrine-committed GFP-negative fraction.

### 3.3. Common Molecules Used During Endocrine Differentiation Elicit Differential Effects on Pancreatic Progenitor Maintenance, Endocrine Differentiation, and Hormone Expression

**Figure 3:**
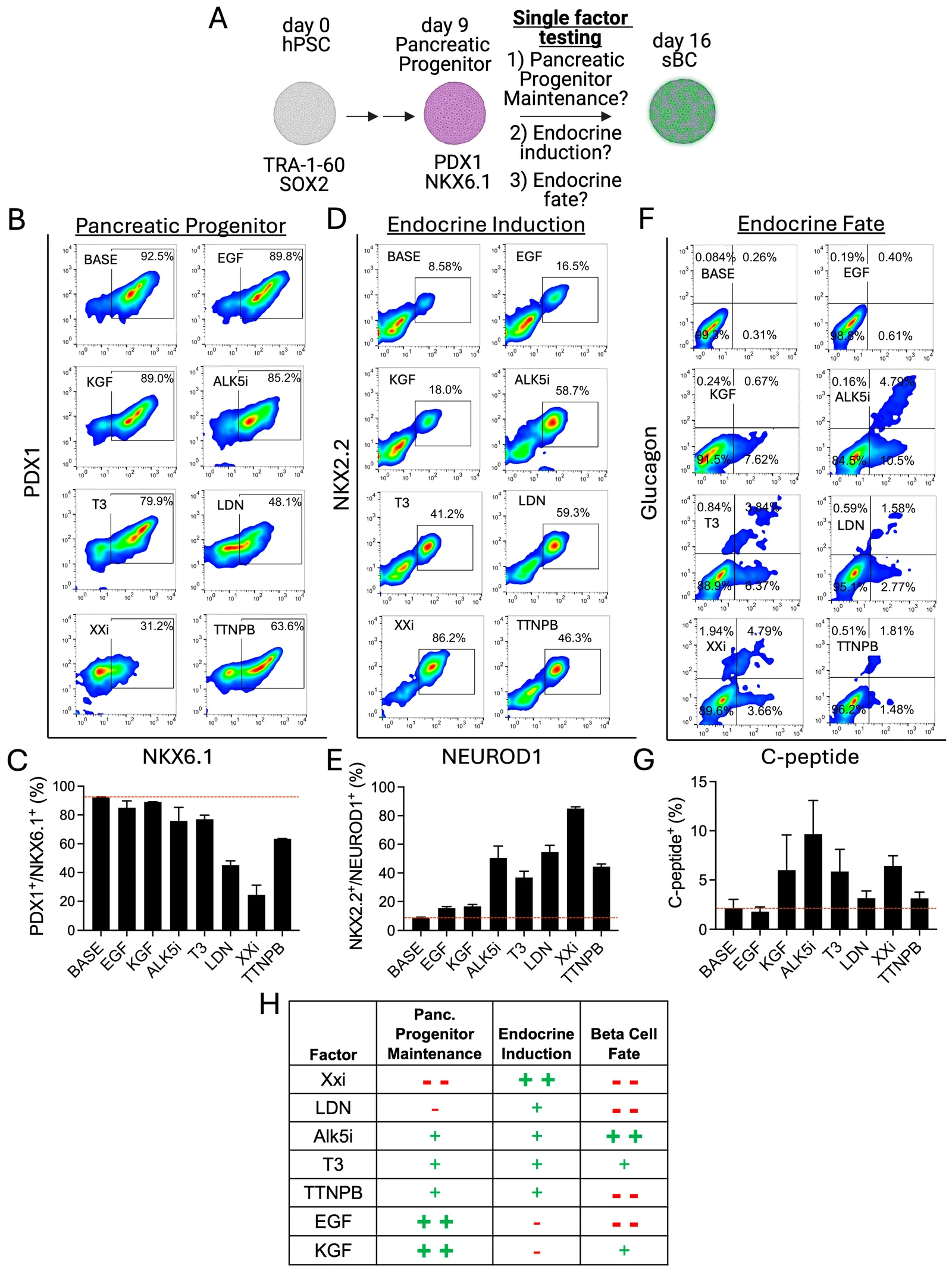
Differential effects of molecules employed during sBC differentiation on progenitor maintenance, endocrine induction and fate. (**A**) Schematic of experimental approach. (**B**–**G**) Representative plots (**top**) and quantification (bottom) of flow cytometric analysis of the percentage of cells expressing the pancreatic progenitor proteins PDX1 and NKX6.1 (**B**,**C**), endocrine differentiation proteins NKX2.2 and NEUROD1 (**D**,**E**) and C-peptide and glucagon proteins (**F**,**G**) after 6 days incubation with control base media only or base media containing denoted single molecules. Red dotted line denotes base media level. Values represent mean with SEM from 2 IDE. (**H**) Schematic representation of the effects of individual factors on endocrine induction and fate determination vs. maintenance of pancreas progenitor identity.

To illuminate the cellular effects of individual molecules used to trigger endocrine differentiation, hESC Mel1^INS-GFP^ clusters were differentiated into pancreatic progenitors (PP) marked by PDX1 and NKX6.1, followed by exposure to the base media of the endocrine cocktail but without any specific molecules added or the same base media containing individual endocrine differentiation factors (Figure 3A). All experimental condition images presented with intact clusters during the incubation period (Supplemental Figure S1A). Employing live imaging of the pINS.GFP reporter revealed that TGFβ inhibition using the ALK5 inhibitor RepSox induced the highest levels of pINS.GFP expression, followed by thyroid hormone T3 and XXi (Supplemental Figure S1A). qPCR analysis further confirmed the observed strong induction of *INS* expression by ALK5i (Supplemental Figure S1B). BMP inhibition using the small molecule LDN-193189 (LDN) and activation of retinoic acid (RA) signaling with the RA analog TTNPB resulted in modest induction of pINS.GFP-expressing cells, whereas the base media alone or with addition of the growth factors EGF or KGF showed only negligible levels (Supplemental Figure S1A). qPCR analysis showed that most factors did not significantly affect *PDX1* or *NKX6.1* expression levels, except for LDN and XXi where a marked reduction in *NKX6.1* gene expression was detected (Supplemental Figure S2B). The PP marker *SOX9* was modestly reduced by all single-factor conditions when compared to the base media control (Supplemental Figure S2B). Notch inhibition via gamma secretase inhibition has been shown to be a critical trigger of pancreatic endocrine differentiation [2,5]. As expected, incubation of PP clusters with XXi alone strongly induced expression of both endocrine markers *NEUROG3* and *NKX2.2* (Supplemental Figure S2B). 

Next, we employed FC analysis to quantify PP markers, PDX1 and NKX6.1, endocrine markers NKX2.2 and NEUROD1, and the hormones insulin (INS) and glucagon (GCG) in each condition. This analysis revealed that EGF, KGF, T3, and TTNPB maintain high levels of PP with double marker expression with a high and medium population present, while the percentage of PP cells was similarly high with ALK5i incubation; however, the high NKX6.1-expresser population was reduced (Figure 3B,C). As anticipated, XXi and LDN revealed lower NKX6.1 protein expression while PDX1 was maintained, validating the RNA expression results. The endocrine markers NKX2.2 and NEUROD1 show that XXi is most effective followed by LDN, TTNPB, ALK5i, and T3 in inducing endocrine marker expression, while EGF and KGF have only a minimal effect on endocrine induction over the base control (Figure 3D,E). These data indicate that XXi and LDN strongly induce endocrine differentiation at the expense of the pancreatic progenitor marker NKX6.1. Interestingly, ALK5i or T3, in contrast, result in the highest induction of insulin on day 16, while only modest expression of hormones was detected with all other molecules, indicating a potentially critical role for early NKX6.1 protein expression in human sBC generation (Figure 3F,G).

Based on these experiments we propose that factors employed in current differentiation protocols exhibit distinct effects on human pancreatic cell types (Figure 3H). Namely, we show that both EGF and KGF maintain high levels of PPs and more importantly NKX6.1 protein expression while preventing endocrine differentiation and subsequent hormone expression. In contrast, XXi and BMPi reduce expression of NKX6.1 but facilitate strong induction of endocrine differentiation, producing mostly glucagon-expressing cells. RA, T3 and ALK5i maintain strong NKX6.1 expression and increasingly induce endocrine differentiation with appreciable generation of insulin^+^ sBCs.

## 4. Discussion

Efficient generation of stem cell-derived β cells (sBCs) requires precise coordination between endocrine commitment and β-cell fate specification, yet these processes remain incompletely understood in human systems. In this study, we dissected both biochemical and physical determinants of endocrine differentiation in 3D culture and identify maintenance of NKX6.1 expression together with controlled aggregate size as key variables associated with β-cell yield and identity during endocrine induction.

Consistent with developmental paradigms, we observed transient induction of the pro-endocrine transcription factor NEUROG3 followed by robust expression of downstream endocrine lineage markers such as NKX2.2, indicating efficient endocrine commitment under standard induction conditions [5,23]. However, despite this high efficiency of endocrine differentiation, only some the cells acquired a β-cell phenotype, and late-stage endocrine-committed, insulin-negative cells failed to convert to sBC upon re-exposure to endocrine differentiation cues. These findings suggest that endocrine commitment and β-cell specification are timely and coordinated processes, and that β-cell fate competence is restricted to a defined developmental window under the culture conditions employed which is consistent with prior observations in hPSC differentiation systems [1,2,3,4,5,6,7,8,9,10,11,12]. 

Our single-factor analysis provides mechanistic insight into this uncoupling. Inhibition of the NOTCH pathway using γ-secretase inhibitors (XXi), and to a lesser extent BMP inhibition, robustly induced endocrine differentiation but resulted in marked downregulation of NKX6.1. While these pathways are effective triggers of endocrine commitment and therefore justified in their widespread use for pancreatic differentiation protocols, they also bias cell fate away from β-cell identity. This observation aligns with extensive in vivo evidence demonstrating a critical role for NKX6.1 in mouse β-cell development, maturation, and functional maintenance [24,25,26]. In contrast, modulation of TGFβ signaling and thyroid hormone signaling preserved NKX6.1 expression while promoting more limited endocrine differentiation and yet resulted in the highest levels of insulin expression. Together, our data suggest that preservation of NKX6.1 protein expression during endocrine induction is a key determinant of human β-cell generation during 3D differentiation.

Although our single factor experiments focused primarily on insulin and glucagon expression, we acknowledge that a more comprehensive analysis of additional endocrine hormones would further refine our understanding of lineage allocation. Such insight is particularly relevant for ongoing efforts to generate stem cell-derived islets containing balanced mono-hormonal expressing populations of β, α, and δ cells [12]. Leveraging the pathway-specific effects defined here, we attempted to shorten the duration of endocrine differentiation during direct differentiation; however, these efforts did not yield significant improvements in β-cell outcomes, even with the addition of ROCK inhibition as previously reported [27]. These results underscore the inherent constraints imposed by developmental timing and fate competence during endocrine differentiation. In parallel with biochemical cues, we identified physical organization as a critical regulator of differentiation efficiency. Initial cluster size exerted a pronounced effect on β-cell yield in 3D culture, with aggregates of approximately 200 μm exhibiting optimal morphology, homogeneity, and insulin-positive cell frequency. This size likely represents a functional optimum governed by competing physical and biological constraints. Below this threshold, insufficient cell–cell contact and limited paracrine signaling may fail to reach the critical mass required to sustain coordinated endocrine specification. Above it, the diffusion limit of oxygen and nutrients in avascular 3D tissue (~>240 μm) risks generating a hypoxic or necrotic core, while steep morphogen gradients may misspecify interior progenitors. Together, these constraints converge on an aggregate size that balances intercellular communication at the periphery with microenvironmental homogeneity throughout the core. These findings are consistent with prior studies demonstrating that 3D architecture and cell clustering strongly influence endocrine maturation and β-cell function in vivo [28].

Importantly, these insights have direct implications for scalability and clinical translation. Small variations in the timing, concentration, or exposure to endocrine induction cues can lead to substantial shifts in cell fate, amplifying heterogeneity during scale-up and compromising product consistency. Moreover, enrichment or purification strategies are difficult to implement at clinical scale, emphasizing the need for differentiation approaches that intrinsically bias cell fate toward β-cell identity rather than relying on post hoc selection. 

## 5. Conclusions

Our data suggest that rational tuning of endocrine induction conditions to preserve NKX6.1 expression, combined with control of 3D hPSC aggregate architecture, offers a path toward more consistent and scalable generation of β-cell products. By identifying NKX6.1 maintenance and aggregate size as important determinants of β-cell identity, our work provides a mechanistic framework for refining endocrine induction strategies and improving the robustness of stem cell-derived β-cell differentiation for both research and translational applications.

## Data Availability

The original contributions presented in this study are included in the article/Supplementary Materials. Further inquiries can be directed to the corresponding author.

## Supplementary Materials

The following supporting information can be downloaded at: https://www.mdpi.com/article/10.3390/cells15171600/s1. Figure S1: Differential effects of factors employed during sBC differentiation on progenitor maintenance, endocrine induction and fate. (A) Bright field and pINS.GFP images of clusters after 6 days incubation with control base media only or base media containing denoted single factor. Scale bar: 200um. (B) qPCR analysis of pancreatic progenitor (PDX1, NKX6.1 and SOX9), endocrine genes (NEUROG3 and NKX2.2) and the hormone insulin (INS) gene expression after 6-day incubation with control base media only or base media containing denoted single factor. Values normalized to GAPDH and shown as mean with SEM from 3 IDE using Mel1 pINS.GFP hESC. One-way ANOVA with multiple comparison test was used to determine statistical significance with * p < 0.05 compared to base condition.; Figure S2: Endocrine marker positive, but insulin negative cells do not induce insulin expression upon re-exposure to published endocrine induction media. (A) Schematic representation of live cell sorting of pINS.GFP- endocrine cells and pINS.GFP+ sBC at day 23 followed by reaggregation and culture for an additional 7 days in endocrine differentiation media. (B) Bright field and fluorescence images of differentiated clusters at day 24 (post-sort), pINS.GFP- endocrine cells and pINS.GFP+ sBC after 1 day in U-bottom plates and after 6 more days of culture in endocrine differentiation media. Scale bar: 100μm. Data representative of 3 IDE; Table S1: List of antibodies and dyes used for flow cytometry; Table S2: List of antibodies and dyes used for immunofluorescence; Table S3: List of primers used for quantitative PCR; Table S4: Diameter; Table S5: Area.
