# Supplementary material for: Physical and Biochemical Determinants of Endocrine Fate in 3D Suspension Culture Based Stem Cell-Derived β-Cell Differentiation
Source: Cells. 2026 Sep 2;15(17):1600. doi: 10.3390/cells15171600 (PMC13564706; doi:10.3390/cells15171600)
Supplement: Supplementary file 1 [file cells-15-01600-s001.zip › cells-4288967-Supplemental.pdf]

Supplemental Information for: Physical and Biochemical Determinants of Endocrine Fate in 3D Suspension Culture based Stem Cell–Derived β-Cell Differentiation
Supplemental Figures:

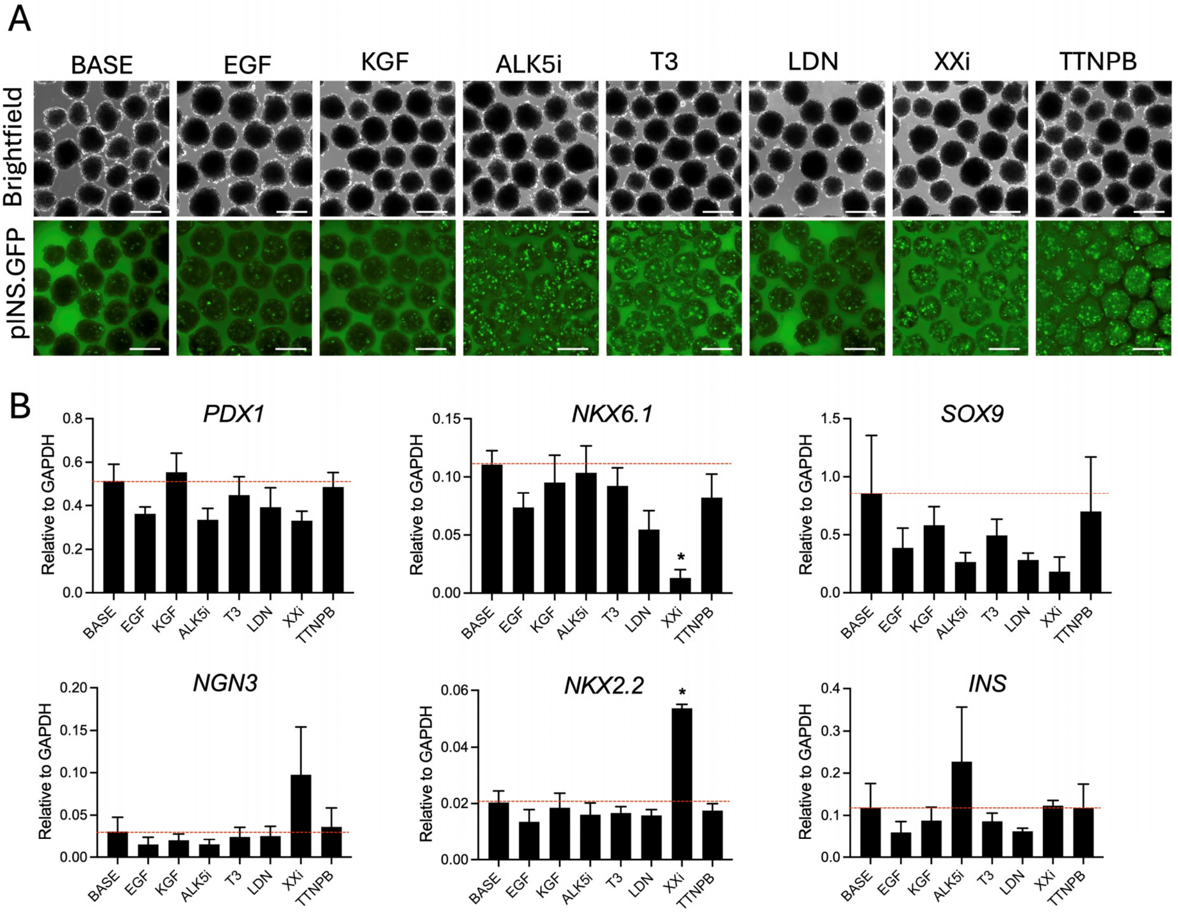

<u>Supplemental Figure S1: Differential effects of factors employed during sBC differentiation on progenitor maintenance, endocrine induction and fate.</u> **(A)** Bright field and pINS.GFP images of clusters after 6 days incubation with control base media only or base media containing denoted single factor. Scale bar: 200um. **(B)** qPCR analysis of pancreatic progenitor (*PDX1, NKX6.1* and *SOX9*), endocrine genes (*NEUROG3* and *NKX2.2*) and the hormone insulin (*INS*) gene expression after 6-day incubation with control base media only or base media containing denoted single factor. Values normalized to *GAPDH* and shown as mean with SEM from 3 IDE using Mel1 pINS.GFP hESC. One-way ANOVA with multiple comparison test was used to determine statistical significance with * $p<0.05$ compared to base condition.

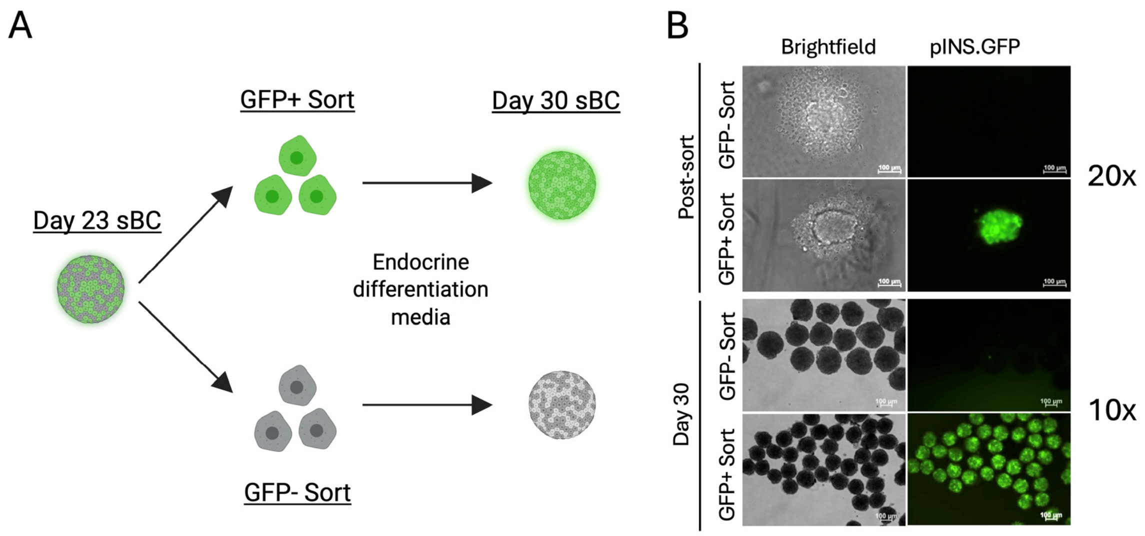

Supplemental Figure S2: Endocrine marker positive, but insulin negative cells do not induce insulin expression upon re-exposure to published endocrine induction media. **(A)** Schematic representation of live cell sorting of pINS.GFP$^-$ endocrine cells and pINS.GFP$^+$ sBC at day 23 followed by reaggregation and culture for an additional 7 days in endocrine differentiation media. **(B)** Bright field and fluorescence images of differentiated clusters at day 24 (post-sort), pINS.GFP$^-$ endocrine cells and pINS.GFP$^+$ sBC after 1 day in U-bottom plates and after 6 more days of culture in endocrine differentiation media. Scale bar: 100µm. Data representative of 3 IDE.

Supplemental Tables:

Supplemental Table S1: List of antibodies and dyes used for flow cytometry

**Antigen**	**Clone**	**Fluorophore**	**Dilution**	**Vendor**	**Catalog #**
C-peptide	C-PEP-01	AF488*	1:300	Origene	BM270
NKX6.1	R11-560	AF647	1:50	BD Biosciences	563338
PDX1	658A5	PE	1:50	BD Biosciences	562161
NKX2.2	74.5A5	PE	1:50	BD Biosciences	564730
NEUROD1	R8.294	AF647	1:50	BD Biosciences	563566
Glucagon	U16-850	BV421	1:500	BD Biosciences	565891

* antibodies were conjugated in house

Supplemental Table S2: List of antibodies and dyes used for immunofluorescence

**Antigen**	**Clone**	**Fluorophore**	**Dilution**	**Vendor**	**Catalog #**
Insulin	Polyclonal	N/A	1:10	DAKO	A0564
NKX-2.2	74.5A5	N/A	1:20	DSHB	74.5A5
NEUROG3	Polyclonal	N/A	1:100	R&D Systems	AF3444
Mouse IgG	polyclonal	AF647	1:500	ThermoFisher	A31571
Goat IgG	Polyclonal	AF555	1:500	ThermoFisher	A21432
Guinea pig IgG	Polyclonal	AF488	1:500	ThermoFisher	A11073

Supplemental Table S3: List of primers used for quantitative PCR.

**Probe**	**Vendor**	**Catalog #**
INS	BioRad	qHsaCPE5192383
NKX6.1	BioRad	qHsaCEP0053372
PDX1	BioRad	qHsaCEP0024751
SOX9	BioRad	qHsaCEP0051345
NGN3	BioRad	qHsaCEP0025582
NKX2.2	BioRad	qHsaCEP0032579
GAPDH	BioRad	qHsaCEP0041396
